# Supplementary material for: Bayesian network predicted variables for good neurological outcomes in patients with out-of-hospital cardiac arrest
Source: PLoS One. 2023 Sep 28;18(9):e0291258. doi: 10.1371/journal.pone.0291258 (PMC10538776; doi:10.1371/journal.pone.0291258)
Supplement: Attachment — Submitted filename: Response to Reviewers.docx [file pone.0291258.s003.docx]

July 13, 2023

Emily Chenette

Editor-in-Chief

*PLoS One*

Dear Editor,

We thank you for considering our paper entitled “Bayesian network predicted variables for good neurological outcomes in patients with out-of-hospital cardiac arrest,” manuscript ID, PONE-D-23-03455, for publication in *PLoS One*. We are hereby re-submitting the revised version of our manuscript.

We are grateful for the feedback provided by the reviewers, which has helped improve the quality of our paper. In accordance with the valuable comments, we have made corrections and additions to the text and figures.

The corrections and additions in the text are highlighted in yellow in the revised manuscript.

We have tried to incorporate your suggestions as much as possible; however, if you have any further suggestions, please let us know.

Thank you for your constructive comments.

Sincerely,

Kota Shinada

Department of Emergency and Critical Care Medicine,

Faculty of Medicine, Saga University

5-1-1 Nabeshima

Saga City, Saga Prefecture 849-8501, Japan

Phone number: +81-952-34-3160

Fax number: +81-952-34-1061

Email address: [st9137@cc.saga-u.ac.jp](mailto:st9137@cc.saga-u.ac.jp)

**Reply to Reviewer 1’s comments**

We appreciate the reviewer’s comments. We have tried to incorporate the reviewer’s suggestions as much as possible, but welcome any additional comments that the reviewer may have.

*The authors discussed about OHCA registry with Bayesian network.*

*To the readers of this paper, I guess that the explanation of machine learning and difference between Bayesian network and deep learning are insufficient.*

*Will this paper have more meanings more than that authors have used Bayesian network.*

*Please explain this point in detail more.*

We thank the reviewer for the suggestion. We have added an explanation in the Introduction regarding the significance of using Bayesian networks in this analysis, as well as a comparison with other deep learning methods (lines 39–53).

**Replies to Reviewer 2’s comments**

We appreciate the reviewer’s comments. We have tried to incorporate the suggestions as much as possible, but welcome any additional comments that the reviewer may have.

*1) the Registry and the methods of data collection should be better described: how many centers participate to the Japan Association for Acute 56 Medicine (JAAM) OHCA registry? What is the population of the area served by the facilities participating to the registry? Are the participating centers able to provide all the necessary treatments to OHCA patients? Please provide a more detailed description of the system as recommended by the Utstein criteria (https://doi.org/10.1161/CIR.0000000000000144) should be provided.*

We thank the reviewer for the suggestion. We have added a statement regarding the number of participating facilities (lines 61–62). However, it was difficult to confirm the population sample for the entire region. The registry includes a variety of hospitals and there may be differences in the care that can be provided. We have also added a statement stating the same in the limitations paragraph (lines 177).

*2) how was the clinical endpoint assessed? Was a study-specific follow-up conducted? or was survival and GCS assessed solely based on health records? How many patients had complete follow-up information?*

We thank the reviewer for the question. There is no follow-up specific to the outcomes of this study. In Japan, one-month survival data is routinely collected in both the Fire and Disaster Management Agency Utstein Registry and Japan Association for Acute Medicine OHCA Registry. GCS and one-month survival was recorded by the respective centers; there were 5 missing cases regarding GCS M (Figure 1) and no cases with missing information on one-month survival.

*3) The description of the Bayesian method should be much more detailed. Was a biostatistician involved?*

We have added a part on the Bayesian network method (lines 46–53). No biostatistician was involved in the study.

*4) It is a major limitation that continuous variables (eg. pH, glucose, creatinine) were categorized into quartiles resulting in cut-offs that have no relationship with validated cut-offs used in clinical activity. Probably a second method should be used to identify predictors of good neurological outcome.*

We thank the reviewer for the suggestion. We have reanalyzed the blood test results by dividing them into categorical variables with clinically relevant values (lines 98–102).

*5) A definition of intrinsic causes of cardiac arrest should be provided. Do the authors mean medical non traumatic causes?*

We thank the reviewer for pointing this out. We have replaced "intrinsic except cardiogenic" with "non-cardiogenic" because extrinsic cardiac arrest was excluded in the first place (Table 1).

*6) The authors should provide the references, on which the selection of the 15 variables was based. It is a major limitation that the variables were selected based on previous literature and authors' judgement.*

We thank the reviewer for pointing this out. We have added some references (line 78) as per the reviewer’s suggestion. We have also mentioned the possibility of bias due to variable selection in the limitations (line 172–174).

*6) The results should be contextualized within the existing literature. In particular the authors should compare the findings of the current analysis with other available risk assessment tools (variables selected, sensitivity and specificity).*

We thank the reviewer for the comment. We have added text on the comparison with previous studies for the selected variables in Table S2 . NULL-PLEASE scores are reported as excellent in previous systematic reviews (Gue YX, et al. Out-of-hospital cardiac arrest: A systematic review of current risk scores to predict survival. Am Heart J. 2021;234:31-41.). We attempted to compare the findings with the NULL-PLEASE score; however, the NULL-PLEASE score included variables that were not collected in the OHCA registry, making actual comparison difficult. We have added the lack of comparison with existing scores to the limitations paragraph (lines 182–184).

*7) Additional information on baseline characteristics and interventions (e.g. CPR provided by emergency medical service, mechanical CPR, coronary angiography, PCI, therapeutic hypothermia), timing of death (KM curves) according to the Utstein criteria (https://doi.org/10.1161/CIR.0000000000000144) should be provided*

We thank the reviewer for pointing this out. We have added as much additional information as we could find: bystander defibrillation, physician-staffed EMS, ECPR, IABP, CAG, PCI, and TTM (Table 1). It was difficult to draw a KM curve for timing of death because of missing data.

*8) English language should be improved. Examples:*

*- Line 99: “with a 100 dominance level set at 5%”: what do you mean?*

*- Line 107: “the patient background information”: i suggest "baseline characteristics and cardiac arrest details"*

*- Line 149: exacerbate the neurological prognosis: "worsen" or "affect" would be more appropriate*

We thank the reviewer for pointing this out.

- We have corrected the first statement to "P<0.05 was considered significant." (line 104-105)

- Corrected as suggested (lines 110–111).

- Corrected as suggested (line 154).

*9) Please provide the measure units of the laboratory values.*

We thank the reviewer for the comment. We have added the unit of measure (Table 1).

*10) Conclusions should be more informative: rather than "age, presence of prehospital adrenaline, GCS M after ROSC, and initial rhythm" i suggest to report older age, absence of prehospital adrenaline, type of rhythm (shockable vs. non shockable).*

We thank the reviewer for the comment. We have revised the conclusions as suggested (lines 25–26 and 189–190)

**Reply to Reviewer 3’s comments**

We appreciate the reviewer’s comments. We have tried to incorporate the suggestions as much as possible, but welcome any additional comments the reviewer may have.

*41-43 Please describe the problems that should be improved in the prognostic and predictive scores of OHCA reported so far. Then, please describe your motivation for conducting this study to address these problems.*

We thank the reviewer for the suggestion. We have added a description in the Introduction (lines 39–53).

*116 What is the basis or criteria for setting the values of No flow time and GCS M cutoffs in Table.1, please describe details in the statistic.*

We thank the reviewer for pointing this out. Blood test items with reference values were divided by the reference value, but variables without reference values, such as the NFT and GCS M, were divided by quartiles. We have added corresponding text in the revised manuscript (lines 98–103).

*151-155 The authors discussed that some cases with ROSC after a relatively short CPR time were included; the prognosis may be better if ROSC does not require adrenaline, and it may also include patients in whom CPR was initiated because they were wrongly assessed to have an OHCA. However, no data are presented to show the relationship between low flow time and adrenaline administration. Also, data are not provided on how many patients were incorrectly evaluated for OHCA.*

We thank the reviewer for the comment. As pointed out by the reviewer, there are no data to support this relationship; therefore, we have deleted the relevant text from the manuscript.

*157-164 The authors discussed that the four variables identified using the Bayesian network may help clinicians in making decisions on the prognosis of OHCA and the overall treatment strategy. Please describe how you would like clinicians to use these four variables to make decisions.*

We thank the reviewer for the advice. We have added a part in the manuscript as per the suggestions (lines 163–169).

*Minor comments*

*67-69 It is better to have a light explanation of the meaning of no flow and low flow at the beginning because it is difficult for some readers to understand.*

We thank the reviewer for the comment. We have added a brief explanation in the revised manuscript (lines 90–92).

*76 Please list citations for the 15 variables.*

We thank the reviewer for pointing this out. We have added citations as suggested (line 78).

*78 Please standardize the terminology to either adrenaline or epinephrine.*

We thank the reviewer for the comment. We have replaced “adrenaline” with "epinephrine" in each instance.
