# Supplementary material for: Bayesian network predicted variables for good neurological outcomes in patients with out-of-hospital cardiac arrest
Source: PLoS One. 2023 Sep 28;18(9):e0291258. doi: 10.1371/journal.pone.0291258 (PMC10538776; doi:10.1371/journal.pone.0291258)
Supplement: Attachment — Submitted filename: Response_to_Reviewers.docx [file pone.0291258.s004.docx]

August 24 2023

Emily Chenette

Editor-in-Chief

*PLoS One*

Dear Editor-in-Chief:

We thank you for considering our paper titled “Bayesian network predicted variables for good neurological outcomes in patients with out-of-hospital cardiac arrest,” manuscript ID, PONE-D-23-03455R1, for publication in *PLoS One*. We are hereby re-submitting the revised version of our manuscript.

We are grateful for the feedback provided by the reviewers, which has helped improve the quality of our paper. In accordance with the valuable comments, we have made corrections and additions to the text and tables.

The corrections and additions in the text are highlighted in yellow in the revised manuscript.

We have tried to incorporate your suggestions as much as possible; however, if you have any further suggestions, please let us know.

Thank you for your constructive comments.

Sincerely,

Kota Shinada

Department of Emergency and Critical Care Medicine,

Faculty of Medicine, Saga University

5-1-1 Nabeshima

Saga City, Saga Prefecture 849-8501, Japan

Phone number: +81-952-34-3160

Fax number: +81-952-34-1061

Email address: [st9137@cc.saga-u.ac.jp](mailto:st9137@cc.saga-u.ac.jp)

**Reply to Reviewer 1’s comments**

*Previous comment is replied appropriately about the explanation of machine learning and difference between Bayesian　network and deep learning. I think that this paper is acceptable.*

Response: Thank you for your confirmation and comments.

**Reply to Reviewer 2’s comments**

*I thank the authors for addressing the majority of my concerns.*

*I have few additional comments:*

Response: Thank you very much for your confirmation and additional constructive comments.

*1) Line 24: I suggest to replace “non-cardiac arrest” with “spontaneous rhythm” or “ROSC”; the label “non-cardiac arrest” as type of rhythm is very confusing.*

Response: We thank the reviewer for the suggestion. We have accordingly made corrections to the lines. (Lines 24, 26, 139, and 196 and tables 1, 2, and S1)

*2) Table 1: I suggest to replace “cardiogenic and non cardiogenic cause” with “cardiac or noncardiac” in agreement with the Utstein guidelines (https://doi.org/10.1161/CIR.0000000000000144)*

Response: We thank the reviewer for the suggestion. We have accordingly made the corrections. (Table 1)

*3) The authors answered to a previous comment: “There is no follow-up specific to the outcomes of this study. In Japan, one-month survival data is routinely collected in both the Fire and Disaster Management Agency Utstein Registry and Japan Association for Acute Medicine OHCA Registry. GCS and one-month survival was recorded by the respective centers; there were 5 missing cases regarding GCS M (Figure 1) and no cases with missing information on one-month survival.”*

*This information should be reported in the methods.*

Response: We thank the reviewer for pointing this out. We have included this in the Methods section. (Lines 72-74)

*4) Please mention in the discussion previous studies that assessed the sensitivity and specificity of other scores, such as the NULL PLEASE*

Response: We thank the reviewer for the suggestion. Citing a systematic review, we have mentioned the predictive performance of the NULL-PLEASE score in the Discussion section. However, it was difficult to mention the sensitivity and specificity as they were not mentioned in most previous studies, although AUC was mentioned.

**Reply to Reviewer 3’s comments**

*The author generally answered all questions somewhat adequately and the arguments were easy to understand.*

Response: Thank you for your confirmation and comments.
